# Validation of the PL-C Quest in China: understanding the pictorial physical literacy self-report scale

**DOI:** 10.3389/fpsyg.2024.1328549

**Published:** 2024-03-07

**Authors:** Yu Wu, Xinxiang Wang, Hongbiao Wang, Lijuan Wang, Ying Tian, Zhiguang Ji, Liyan Wang

**Affiliations:** ^1^College of Rehabilitation, Shanghai University of Medicine & Health Science, Shanghai, China; ^2^College of Sports Science, Shenyang Normal University, Shenyang, China

**Keywords:** physical literacy, measurement, child, self-report scale, cultural adaptation

## Abstract

**Introduction:**

The notion of physical literacy is gaining interest from several countries as a potential mechanism for understanding the development of the physical self. This research endeavor represents an inaugural attempt to translate the Australian Physical Literacy Questionnaire for Children (PL-C Quest) into Chinese to evaluate the reliability and validity of the Chinese version of the PL-C Quest to assess physical literacy among children in mainland China.

**Methods:**

The Beaton translation paradigm was used to carry out language translation, back-translation, cultural adaptation, and presurveys. Data were collected from 642 children aged 6–12 years, with a mean age of 9.71 years (SD 1.816), to test the reliability of the Chinese version of the PL-C Quest.

**Results:**

The PL-C Quest items translated well (6.187 ~ 15.499) and correlated well (0.441 ~ 0.622). The Chinese version of the PL-C Quest had good reliability, with retest reliability values ranging from 0.91 to 0.74, Cronbach’s alpha from 0.65 to 0.894, and McDonald’s ω from the Spearman-Brown Coefficient was 0.84. The validity results are acceptable because the CFI, IFI, and TLI values are above 0.8 and close to 0.9, but the model fit’s chi-square degrees-of-freedom ratio of 2.299, the RMSEA of 0.05, which was less than 0.08.

**Discussion:**

After translation and cultural adaptation, the Chinese version of the PL-C Quest is a reliable measurement tool and can be used in the Chinese region.

## 1 Introduction

In recent years, physical literacy has emerged as an elaborate understanding of why and how to get children moving (Barnett et al., 2022). Physical literacy is defined as “the motivation, confidence, physical competence, knowledge, and understanding to value and take responsibility for engagement in physical activities for life” (Stevens, 2016). Research indicates that physical literacy is associated with body composition, physical fitness, blood pressure, and health-related quality of life (Caldwell et al., 2020) but also serves as a significant indicator of an individual’s future physical and mental well-being (Bailey, 2022). In the case of children, the level of physical literacy can reflect their potential for engaging in lifelong physical activity, as well as the effectiveness of school physical education and health education (Kleis et al., 2022). Physical literacy assessment is a doctrinal necessity for developing a comprehensive assessment tool for youth sports, and its administration helps to determine the level of children’s physical literacy, which, in turn, supports the development of children’s physical literacy. Hence, the assessment and evaluation of children’s physical literacy level is presently a prominent area of research (Kleis et al., 2022), with scholars from different nations having devised multiple assessment instruments for this purpose (Bremer et al., 2019; Shearer et al., 2021).

There are two notable characteristics associated with current physical literacy evaluation systems. The assessment primarily relies on the combination of objective and subjective evaluation methods (Hulteen et al., 2020). These methods include the use of Canadian assessment physical literacy (CAPL-2) (Longmuir et al., 2015), Passport for Life (P4L) (Lodewyk, 2019), and PLAYfun (Stearns et al., 2018). The exam consists of three components: a tracking test, a questionnaire, and a field test. On the other hand, the measurements were mainly subjectively evaluated through scales or questionnaires with textual descriptions, such as the Perceived Physical Literacy Inventory (PPLI) (Sum et al., 2018) Adolescent Physical Literacy Questionnaire (APLQ) (Mohammadzadeh et al., 2022). However, these assessment tools have certain limitations. The text version of the scales lacks justification for the comprehension of children of younger ages (Alain, 1996). Objective assessment tools are challenging to help children understand the meaning of the assessment and cause questions and uncertainty (Baxter et al., 2011). These assessments do a poor job of encouraging children to develop self-awareness through self-evaluation (Hooker et al., 2012). Physical literacy refers to the amalgamation of physical and mental aspects, some of which can be objectively assessed, such as the use of specific instruments to measure athletic prowess (Hulteen et al., 2020). Other aspects involve the psychological dimension, such as the motivation to engage in sports, and elements of an individual’s emotional connection to sports, which necessitate self-reporting for measurement and evaluation (Longmuir et al., 2017; O’Halloran et al., 2018).

In this regard, the Physical Literacy in Children Questionnaire (PL-C Quest), developed by Sport Australia, is one of the most important initiatives in children’s physical literacy (Australia Sport 2021). The assessment is based on a “pictorial” self-report format that is most closely related to the concept of perceived physical literacy in children. The questionnaire questions are presented in the form of pictures and children self-report their level of physical literacy (Longmuir et al., 2017). Self-reports are evaluations made after self-observation of the physical state of own self, thoughts and behaviors. Existing studies have shown that there is consistency between self-reported physical activity levels and true physical activity (Harter and Pike, 1984; Hulteen et al., 2020). However, text versions of self-report scales or questionnaires are clearly not user-friendly for children with limited literacy. Children’s pictorialized scales have been developed and used in assessment domains such as Fundamental Movement Skill (FMS) (Alain, 1996; Baxter et al., 2011; Filho et al., 2021), which accurately addresses the distress caused by reading text. Numerous studies have demonstrated (Harter and Pike, 1984; Alain, 1996; Baxter et al., 2011; Hooker et al., 2012; Filho et al., 2021) that such scales can effectively address the inability of younger children to read accurately on their own.

China’s existing physical literacy assessment tools Chinese Assessment and Evaluation of Physical Literacy (CAEPL) (Chen et al., 2020) and Perceived Physical Literacy Inventory (PPLI) (Sum et al., 2018) are both presented in textual form, targeting a large span of the population, with relatively low applicability to children aged 4–12 years (Hooker et al., 2012). Compared with Western countries, China has not yet formed a physical literacy system for the whole life cycle of human beings (Li et al., 2022), and most of the studies mainly focus on the high school education stage, with less guidance for the development of primary and secondary school students and other age groups (Sum et al., 2020). In addition, using self-reports based on children’s assessments is relatively rare in China, and using assessments that incorporate images is even rare (Li et al., 2022). Therefore, learning from PL assessment tools in physical literacy building from one of the more developed countries is essential.

In this study, the PL-C Quest will be Chineseized to address the lack of reading autonomy in younger children with a pictorial self-report measure and to cultivate Chinese children’s ability to perceive physical literacy while assessing children’s level of physical literacy in a relatively authentic, accurate, and comprehensive manner. The use of PL-C Quest can highlight the subjective position of children in physical literacy assessment, emphasize children’s self-perception and self-evaluation of physical literacy, and better focus on children’s subjective feelings and sensations (Australia Sport 2021). Not only that, children with higher levels of perception showed stronger self-esteem (Barnett et al., 2015). They were willing to put in more effort and choose challenging tasks, which motivated children to challenge lifelong physical activity (Lopes et al., 2016). In order to achieve these goals, a population of children aged 6–12 years old in China was recruited for this study and revised into an assessment tool of physical literacy for use with Chinese children. To provide the feasibility of the PL-C Quest in the Chinese cultural context and to compare the existing evidence of children’s self-perceived physical literacy to become a validated tool to validate the level of children’s physical literacy globally.

## 2 Methods

### 2.1 Participants

This study was an offline public survey (advertised as a study of children’s physical literacy) conducted from November 2022 to July 2023. The inclusion criteria for the study population were school students of 660 healthy children aged 6 to 12 years, and the exclusion of non-school students ensured that the sample was representative. Socio-demographic data were collected, including name, gender, age, region (see Table 1).

**Table 1:** Demographic characteristics (*n* = 642).

Demographic characteristics	Presurvey	Test–retest	Reliability test
Number	20	30	642
Boys	7 (35%)	15 (50%)	341 (53.1%)
Girls	13 (65%)	15 (50%)	301 (46.9%)
Age/M	8.45	9.48	9.71
Age/SD	2.164	2.064	1.816
6–8 years old	100 (100%)	13 (43.3%)	229 (35.6%)
9–12 years old	100 (100%)	17 (56.7%)	413 (64.4%)
North			65 (10.1%)
Central Northeast	20 (100%)		110 (17.1%)
East Coast		30 (100%)	316 (49.2%)
Northwest			151 (23.5%)

Demonstrates the overall sample distribution of demographic information and percentage statistics for sample inclusion, including M (mean), SD (standard deviation).

Sample 1 was analyzed as a pre-survey to test whether the scale was consistent with Chinese children’s routine answering questions, including younger children (*n* = 10) and older children (*n* = 10)—an elementary school in central northeastern China for the March 9, 2023 distribution. Twenty valid questionnaires with a mean age of 8.45 (SD 2.164) were collected from 7 boys and 13 girls.

Sample 2 To validate the scale retest reliability, 30 children (boys 50%) with a mean age of 9.48 (SD 2.064) were randomly recruited from an elementary school on the east coast of China. Sample 2 gathered on March 23, 2023, 12 days apart from the second distribution.

Sample 3 to test the reliability and validity of the scale. Distribution was conducted from March 9, 2023, to July 18, 2023, using simple sampling from four elementary schools on the east coast, north, central northeast, and northwest China. The number of valid data collected by adding the data from Sample 1 and Sample 2 is 642, with a mean age of 9.71 years (SD 1.816), including 53.1% (*n* = 341) boys and 46.9% (*n* = 301) girls. According to Kendall’s sample size calculation, the criteria of 22:1 was achieved, and the strict ratio of 20:1 according to the current study (Hogarty et al., 2005), which is also adequate.

### 2.2 Ethics approval

The study involving human participants were reviewed and approval by the Ethics Committee at Shanghai University of Medicine and Health Science (protocol code: 2023-PEEC-10-150203198809134265).

Prior to the start of the study, all principals, teachers, and parents/guardians received written information about the study, after which the legal guardians of all participants were invited to sign an informed consent letter. The tester will also read the informed consent form and the test before the child responds, and the child can opt out of the program at any time if he/she himself/herself declines to participate.

### 2.3 Measure and procedure

**Figure 1:**
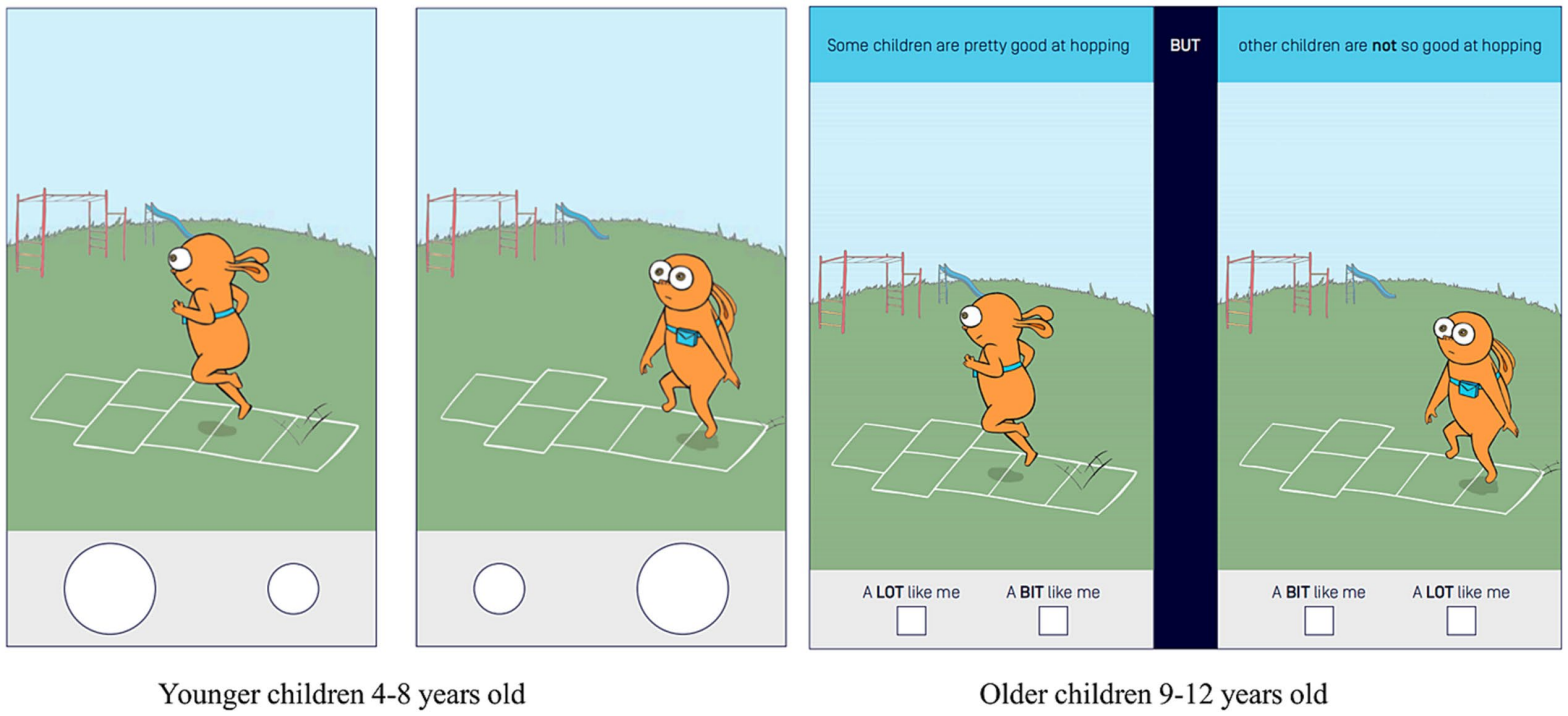
Younger children version and older children version.

The PL-C Quest was developed by the Australian Sports Commission and Prof. Lisa M. Barnett’s team based on the Australian Physical Literacy Framework (APLF). Copyright for using the Australian Sports Commission (ASC) PL-C Quest was obtained before this study (see Supplementary material S1). The questionnaire consists of four sections: physical, psychological, social, and cognitive with a total of 30 questions. It is divided into two versions for older children and younger children. Younger children (4–8 years old) and older children (9–12 years old) were tested using different versions but the same content questionnaire (subtle differences in having only pure pictures vs. both picture text, see Figure 1). Each question of the scale included two pictures with an orange-colored rabbit as the main character. Figure 1 shows that each scenario in the scale corresponds to two conditions, one for the rabbit showing better physical literacy and one less so (Australia Sport 2021).

Before the official survey, professional surveyors (mostly teachers specializing in education) are trained and familiarized with test manuals. The questionnaire instructor will provide appropriate explanations for questions that the children cannot answer or have difficulty answering but will not intentionally guide the children to answer a particular aspect of the question. The children themselves should fill in all the answers to ensure that the information collected by the questionnaire is objective and accurate.

The younger children version is administered to children between the ages of approximately 4 and 8 years old, and this version does not contain any text. Thus, it is administered in a “face-to-face” format, with the teacher or evaluator instructing each child individually to complete the questionnaire (see Figure 1).

The older children version respondents are approximately 9 to 12 years old. Children in this age group can read and write well enough to complete the questionnaire on their own under the guidance of an assessor.

If the child chooses the image on the left that he/she is better at, he/she receives a score of 4 (A LOT like me) or 3 (A BIT like me), and if the child chooses the image on the right that he/she is less good at, he/she receives a score of 2 (A LOT like me) or 1 (A BIT like me) (see Figure 1).

### 2.4 Quality control

To ensure the completeness of the data, the basic situation of the child questionnaire was required to be filled out in clear and legible handwriting, with no obvious logical errors and no missing items. A total of 660 people were recruited, 642 data were included after quality control, and 18 unqualified data were excluded.

### 2.5 Translation

English language PL-C Quest materials were culturally adapted and translated according to a method previously described by Beaton (Beaton et al., 2000). It was divided into five steps (1) translation, (2) synthesis, (3) back-translation, (4) expert review, and (5) prediction.

In the first and second steps, the original PL-C Quest questionnaire was translated by two translators who independently completed the preliminary translation work, and two preliminary translations (a and b) were formed after the translation was completed.

Third, two bilingual graduate students who had not been exposed to the original scale and were more familiar with the field of kinesiology were invited to translate the Chinese version of the Physical Literacy Scale back into English.

Fourth, physical education experts who were familiar with the field related to the content of the study and had rich experience in foreign cultural exchange were invited to conduct comprehensible analysis and cognitive interviews on the questionnaire, as well as all the researchers who participated in the translation.

**Figure 2:**
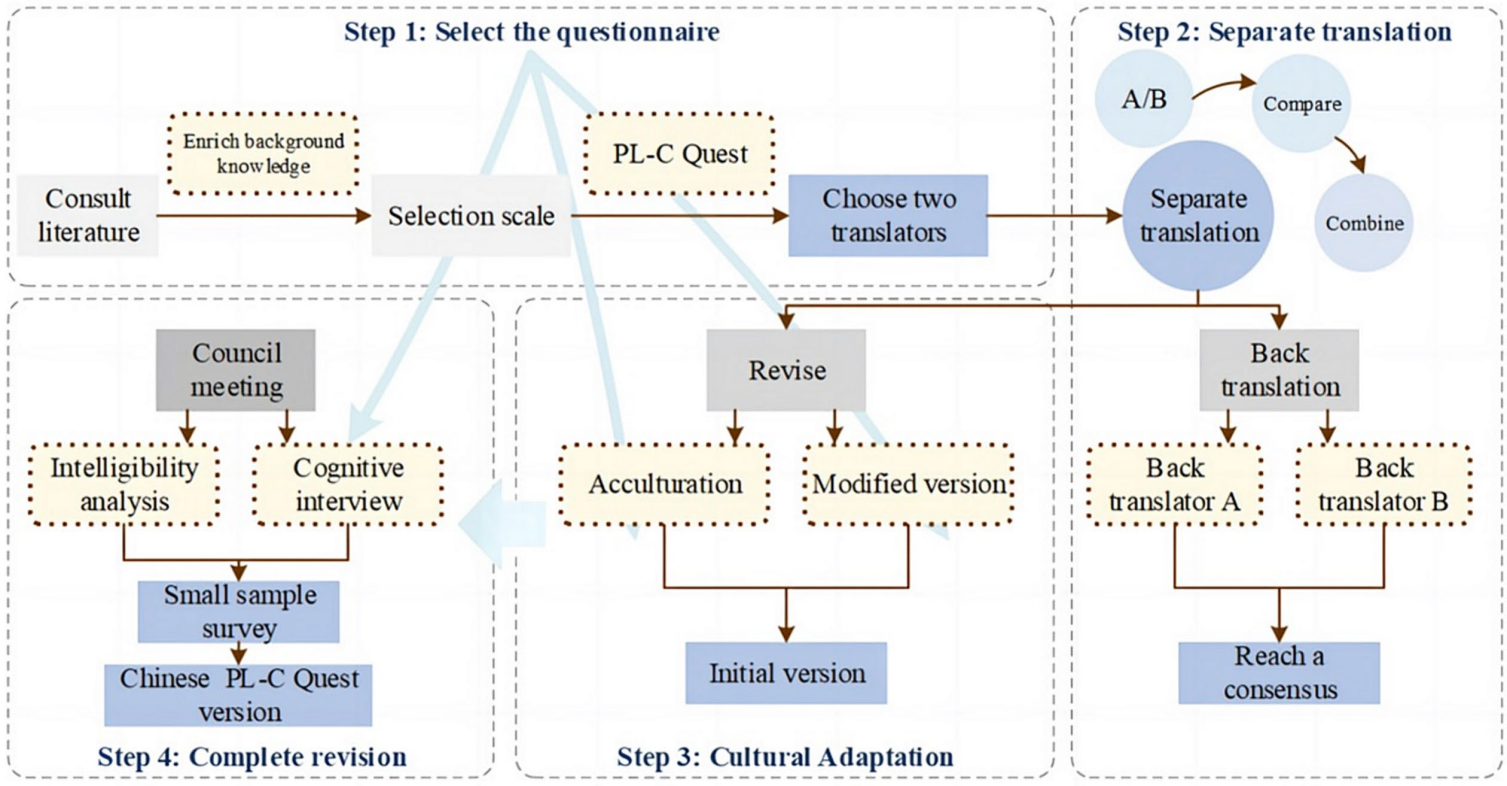
Procedure for translation and culture adaptation of the Chinese version of the questionnaires belonging to the PL-C Quest.

Fifth, 20 children were selected for presurvey testing to form the Chinese version of the physical literacy questionnaire (see Figure 2).

### 2.6 Statistical analyses

Descriptive analysis of the sample characteristics (gender, age, region, and grade) was first performed using IBM SPSS 27 (Chicago, IL). The sample was then subjected to the Kolmogorov–Smirnov test, and the variables obeyed an average distribution skewness <1.19 and kurtosis<1.96 (Nolan and Heinzen, 2011). Afterward, the critical ratio method (>3 discrimination is excellent) and the total question correlation method (*p* < 0.001 correlation is excellent) was used to test the correlation as well as the discrimination of the items (Xie et al., 2022). Reliability tests were performed using internal consistency reliability (Mokkink et al., 2010), split-half reliability (Zhang and Yuan, 2016), and McDonald’s omega (all >0.7 is excellent) (Landis and Koch, 1977). The intragroup correlation coefficient (ICC) was then assessed by calculating the retest reliability, and at the 95% confidence interval, an ICC of 0.61 to 0.80 was considered good; >0.80, almost perfect (Landis and Koch, 1977).

The structural validity of the scale was analyzed using IBM SPSS Amos 26.0 software. Structural validity was assessed using validated factor analysis (CFA). Model fitting indicators used the chi-square degrees of freedom ratio χ^2^/df, RMSEA, CFI, IFI, and TLI. The excellent range of chi-square degrees of freedom ratio χ^2^/df was within 1–3; RMSEA in the range of <0.05 was excellent; and the coefficient values of CFI, IFI, and TLI indicators >0.85 were at the acceptable level (Hu and Bentler, 1999). Because PL-C Quest each item correlation is relatively strong, there may be lower factor loading (Barnett et al., 2022), this study draws on the minimum requirement of >0.4 as a criterion (Farrés-Tarafa et al., 2022).

## 3 Results

### 3.1 Cognitive interview with the Chinese PL-C Quest

There are professional translators and graduate students in sports science who translated the Chinese version of the PL-C Quest program. After this, 10 panels of psychological and sports psychology experts were invited to evaluate the program. They were made explicit about the definition of physical literacy and then asked to evaluate each item with two questions: (1) “Is the items clear?” and (2) “In your opinion, does the items reflect the physical literacy described? These two questions were rated on a Likert scale ranging from 1 (strongly disagree) to 10 (strongly agree). In addition, there was an open-ended question (“Do you have any concerns about the items written? If so, what concerns do you have?”), so that they could express their concerns as needed.

These 10 evaluators’ assessments indicated that the items were clear and reflected the physical literacy structure and the scores of these 10 evaluators ranged from 7.17 to 9.33. They also raised some questions, with one participant stating: “I would like to know if some children are not able to move forward quickly when they hear the gun at the start, is it because they are not physically able to move quickly enough or because they cannot react quickly enough? Perhaps the item should read: “Some children do not react quickly to the gunshot at the start.” Another suggestion is to change “pretty good” to “could” in some scenes. In China, we seldom use the expression “pretty good,” but more often, “I can do it.” For example, “Some children are pretty good at running for a long time without getting tired” has been changed to: “Some children can run for a long time without getting tired.”

After doing so, we randomly selected 20 elementary school students between the ages of 6–12 years old for the questionnaire presurvey. The result feedback was that there was no objection to fill in the questionnaire of the older children version, and it could be assessed in the form of words and pictures. However, during the testing process, we found that children aged 6–8 years old have certain literacy skills, and some topics can be understood and filled out based on pictures combined with words. Since we did not find 4–5-year-old subjects, we were unable to make further determinations on whether to change the age range of the older child version to 6–12 years old.

### 3.2 Preliminary analyses

Epi Data 3.1 software was used to select two people to enter data independently and create a database, while the data were logically checked and randomly sampled using a computer to ensure the accuracy of the data. Descriptive statistics, including score means, scoring distribution, skewness, and kurtosis, are reported in Table 2. The absolute skewness of the four dimensions of the scale (all less than 1.19) but kurtosis showed that the social domain showed a sharp peaked state (platykurtic) as compared to the cognitive domain. The normal distribution was short peaked and thin tailed (less peak and thin tail) suggesting that the distribution of means was spread out, with low extremes and lower risk (Nolan and Heinzen, 2011). Total scale scores were significantly correlated. The correlations between the dimensions and the total score ranged from 0.452 to 0.701. A *t*-test revealed no significant gender differences for the four domains (*p* > 0.05) or the total score (*t*(642) = 2.701, *p* > 0.001).

**Table 2:** Subscale descriptive and scoring distribution for PL-C Quest (Chinese) (*N* = 642).

PL-C Quest domains and items (%)	Girls						Boys						Total						Girls vs. Boys	
	point1	point2	point3	point4	Mean/SD	Mid	point1	point2	point3	point4	Mean/SD	Mid	point1	point2	point3	point4	Mean/SD	Mid	S/K	*p* value
Sum of Domains (30 items) Range 30–120					95.13/14.57	97					98.25/14.6	100					96.76/14.69	98		
Physical (12 items) Score Range 12–48					35.99/6.76	36					37.44/6.65	38					36.76/6.74	37	S:-0.56 K:0.428	
Movement skills	6.3	11.6	30.9	51.2			7.9	12.9	23.8	55.4			7.2	12.3	27.1	53.4				
Moving using equipment	33.6	29.2	17.6	19.6			41.6	18.8	12.3	27.3			37.9	23.7	14.8	23.7				
Object manipulation	12.6	15.6	31.2	40.5			6.5 s	9.1	20.8	63.6			9.3	12.1	25.7	52.8				**<0.001**
Coordination	17.3	20.6	26.6	35.5			15.5	16.1	17.6	50.7			16.4	18.2	21.8	43.6				
Stability/balance	17.3	22.6	30.6	29.6			19.9	19.9	23.5	36.7			18.7	21.2	26.8	33.3				
Flexibility	8.6	12	31.2	48.2			14.1	10.6	23.2	52.2			11.5	11.2	26.9	50.3				
Agility	7	9.6	21.9	61.5			7	7.6	20.2	65.1			7	8.6	21	63.4				
Strength	10	11.3	22.9	55.8			21.7	14.4	19.6	44.3			16.2	12.9	21.2	49.7				
Muscular endurance	14	15	28.9	42.2			9.7	9.7	23.5	57.2			11.7	12.1	26	50.2				
Cardiovascular endurance	8.6	13	32.6	45.8			6.2	5.9	22.3	65.7			7.3	9.2	27.1	56.4				**<0.001**
Reaction time	9	15.6	33.9	41.5			5	11.4	23.2	60.4			6.9	13.4	28.2	51.6				**<0.001**
Speed	14	16.9	31.9	37.2			12.9	13.2	22.6	51.3			13.4	**15**	26.9	44.7				
Psychological (7 items) Score Range 7–28					22.33/4.213	23					23.4/4.06	24					23/4.15	24	S: −0.98 K:1.176	
Engagement enjoyment	11.3	15.3	31.6	41.9			7.9	11.1	26.4	54.5			9.5	13.1	28.8	48.6				
Confidence	6	6	32.6	55.5			7.3	8.5	23.2	61			6.7	7.3	27.6	58.4				
Motivation	4.7	6	27.9	61.5			4.1	6.5	21.1	68.3			4.4	6.2	24.3	65.1				
Connection to place	10	14.3	26.6	49.2			11.7	9.7	25.8	52.8			10.9	11.8	26.2	51.1				
Self-perception	20.6	16.9	20.6	41.9			16.4	10.3	18.8	54.5			18.4	13.4	19.6	48.6				
Self-regulation (emotions)	7	12.3	30.9	49.8			7.6	7.6	23.5	61.3			7.3	9.8	26.9	55.9				
Self-regulation (physical)	6	6.6	23.3	64.1			5	6.7	20.5	67.7			5.5	6.7	21.8	66				
Social (4 items) Score Range 4–16					13.61/2.408	14					13.7/2.55	14					13.66/2.48	14	S: −1.37 K:2.042	
Relationships	9.3	14.3	33.9	42.5			10.9	12	24.9	52.2			10.1	13.1	29.1	47.7				
Collaboration	4	6	25.3	64.8			4.7	5	19.4	71			4.4	5.5	22.1	68.1				
Ethics	2.7	6.6	23.6	32.9			5.6	3.5	16.4	74.5			4.2	5	19.8	71				
Society & culture	4	6	30.2	59.8			7.3	9.1	24.6	58.9			5.8	7.6	27.3	59.3				
Cognitive (7 items) Score Range 7–28					22.97/3.685	23					23.71/3.95	24					23.36/3.84	24	S:-1.035 K:1.928	
Content knowledge	5.3	5	26.9	62.8			5.3	4.1	18.5	72.1			5.3	4.5	22.4	67.8				
Safety & risk	8.6	19.9	32.6	61.6			10	10	31.1	49			9.3	14.6	31.8	44.2				
Rules	2	4.7	16.6	76.7			6.7	4.1	15.2	73.9			4.5	4.4	15.9	75.2				
Reasoning	11	12.3	33.6	43.2			13.8	10.6	22.9	52.8			12.5	11.4	27.9	48.3				
Strategy & planning	7	8.3	26.2	58.5			9.7	7	25.2	58.1			8.4	7.6	25.7	58.3				
Tactics	14	18.3	33.2	34.6			8.8	6.5	21.7	63			11.2	**12**	27.1	49.7				**<0.001**
Perceptual awareness	5.6	5.6	25.6	63.1			5	5.9	20.2	68.9			5.3	5.8	22.7	66.2				

The scores of 642 children (boys *n* = 341 and girls *n* = 301), the four dimensions, and 30 items of the PL-C Quest, including M (mean), SD (standard deviation), Mid (middle) S (skewness), and K (kurtosis) for each domain. The bolded p-values represent that significantly different in the boys and girls’ items.

The score range for the physical domain is 12 to 48, the social domain is 4 to 16, and both the psychological and cognitive domains are 7 to 28. Table 2 summarizes the self-ratings of children’s physical literacy levels in China (each question corresponds to a score of 1–4). The highest and lowest scoring items in the children’s physical literacy level are listed by percentage of the number of people and categorized by gender. The mean of Chinese children’s self-assessed physical literacy level is 96.76. While there are no gender differences between overall scores and individual domains. However, four items demonstrated significant gender differences (*p* < 0.001), namely object manipulation, cardiovascular endurance, Reaction time in the physical domain, and Tactics in the cognitive domain. Within the physical domain, the children’s highest self-ratings (point 4) were agility (63.4), and the lowest (point 1) was moving using equipment (37.9). The highest self-rated item by children in the psychological domain was self-regulation physical (66), and the lowest was self-perception (18.4). The highest-scoring item in the social domain was Ethics (71), and the lowest was Relationships (10.1). The highest-scoring item in the cognitive domain was rules (75.2), and the lowest was reasoning (12.5).

### 3.3 Total item analysis

We performed the critical ratio method with the question total correlation method on the PL-C Quest questionnaire. First, we used the critical ratio method to calculate the total score from the PL-C Quest scores, grouping the top 27% of total scores (*n* = 173) with the bottom 27% of total scores (Xie et al., 2022), followed by independent samples t-tests to analyze to analyses the differences between the two groups on individual questions. This was followed by a question-total correlation method, where the total scale score was calculated and then correlation analysis was performed with the individual scale questions.

The CR values for the 30 items of the PL-C Quest ranged from 6.187 to 15.499, which were all greater than 3.0, and were differentiated between the high and low scoring groups for each question item (*p* < 0.01). This indicates that the 30 question items were clearly differentiated and the study retained all of them. In addition, the results of the specific analyses showed that the correlation coefficient values ranged from 0.441 to 0.622, and the correlation coefficients between the items and the total score were all greater than 0.4, indicating that the correlation between the individual items and the total score of the scale was high (Supplementary Table S1).

### 3.4 Reliability

Internal consistency reliability was assessed based on Cronbach’s alpha (Mokkink et al., 2010). The results indicated that the PL-C Quest had good internal consistency reliability (*α* = 0.894). For the Social domain, Cronbach’s alpha was acceptable (*α* = 0.652), indicating normal internal consistency. After that, McDonald’s ω coefficient was used to test the factor analysis of each question to condense the information, *ω* = 0.903, each question item has excellent reliability; then the 30 questions were divided into two groups using split-half reliability for validation, and the value of Spearman-Brown Coefficient was 0.84, specific information is given in Table 3.

**Table 3:** McDonald’s *ω* and split-half reliability (*n* = 642).

Items	McDonald’s ω	Items	McDonald’s ω	Total	Guttman
1	0.901	16	0.9	0.903	0.839
2	0.901	17	0.899		
3	0.901	18	0.898		
4	0.899	19	0.9		
5	0.9	20	0.899		
6	0.901	21	0.9		
7	0.901	22	0.9		
8	0.903	23	0.899		
9	0.901	24	0.899		
10	0.899	25	0.899		
11	0.898	26	0.902		
12	0.898	27	0.898		
13	0.898	28	0.9		
14	0.899	29	0.899		
15	0.899	30	0.9		

Demonstrates the items of McDonald’s ω and Guttman Split-Half Coefficient totals.

Finally, 30 elementary school students (50% boys) aged 6–12 years old were randomly selected to retest the reliability of the older children’s version (*n* = 17) and the younger children’s version (*n* = 13). Each subject was invited to the classroom twice for a total of 2 questionnaire responses, 12 days apart. Table 4 shows in detail how each dimension and the total score were filled in for the overall, older and younger children, and it is seen that the overall retest reliability value was excellent (ICC = 0.91 confidence interval (CI) [0.81, 0.96]). Among the domains, the intergroup coefficients for the social domain were moderate (ICC = 0.74 [0.45, 0.88]), followed by the cognitive domain (ICC = 0.75 [0.48, 0.88]). The psychological domain (ICC = 0.87 [0.73, 0.94]) had a high between-group coefficient with the physical domain (ICC = 0.93 [0.85, 0.97]). We found that younger children had higher overall within-group coefficients than older children (0.95 vs. 0.90), as well as higher physical (0.85 vs. 0.73) and cognitive (0.88 vs. 0.69) coefficients than older children. However, younger children had lower within-group coefficients for psychological (0.82 vs. 0.89) and social (0.68 vs. 0.74) factors than older children. The comprehensive validation showed that this questionnaire has good reliability.

**Table 4:** Retest reliability for older vs. younger children (*n* = 30).

Physical literacy domains		Test 1				Test 2				Test-retest reliability		
		Min	Max	M	SD	Min	Max	M	SD	ICC	95% LCI	95% UCI
Sum of Domains (30 items) Range 30–120	All	61	118	94.97	16.53	61	118	95.27	16.22	0.91	0.81	0.96
	6–8	69	118	97.92	13.89	68	118	95.00	13.74	0.95	0.85	0.98
	9–12	61	118	92.71	18.38	61	118	95.47	8.30	0.90	0.73	0.96
Physical (12 items) Score Range 12–48	All	12	46	35.67	7.85	16	48	36.07	7.38	0.93	0.85	0.97
	6–8	12	46	35.69	9.21	16	46	34.77	8.08	0.95	0.86	0.98
	9–12	23	46	35.65	6.94	22	48	37.05	6.86	0.91	0.75	0.96
Psychological (7 items) Score Range 7–28	All	10	28	23.07	4.18	9	28	22.47	4.90	0.87	0.73	0.94
	6–8	17	28	24.00	3.14	16	28	22.76	4.12	0.82	0.41	0.94
	9–12	10	28	22.35	4.80	9	28	22.23	5.53	0.89	0.71	0.96
Social (4 items) Score Range 4–16	All	6	16	13.60	2.66	7	16	13.37	2.98	0.74	0.45	0.88
	6–8	12	16	14.30	1.49	10	16	13.76	2.04	0.68	0.44	0.90
	9–12	6	16	13.05	3.23	7	16	13.05	3.56	0.74	0.29	0.90
Cognitive (7 items) Score Range 7–28	All	11	28	22.63	4.14	17	28	23.37	3.34	0.75	0.48	0.88
	6–8	19	28	23.92	3.20	19	28	23.69	3.03	0.88	0.61	0.96
	9–12	11	28	21.65	4.58	17	28	23.11	3.62	0.69	0.15	0.88

Table 2 reports the test–retest scores for the four domains for the entire children (younger children and older children) with the minimum, maximum, M (mean), SD (standard deviation), between-group coefficients, and lower confidence interval (LCI); upper confidence interval (UCI). upper confidence interval (UCI).

### 3.5 Confirmatory factor analysis

**Figure 3:**
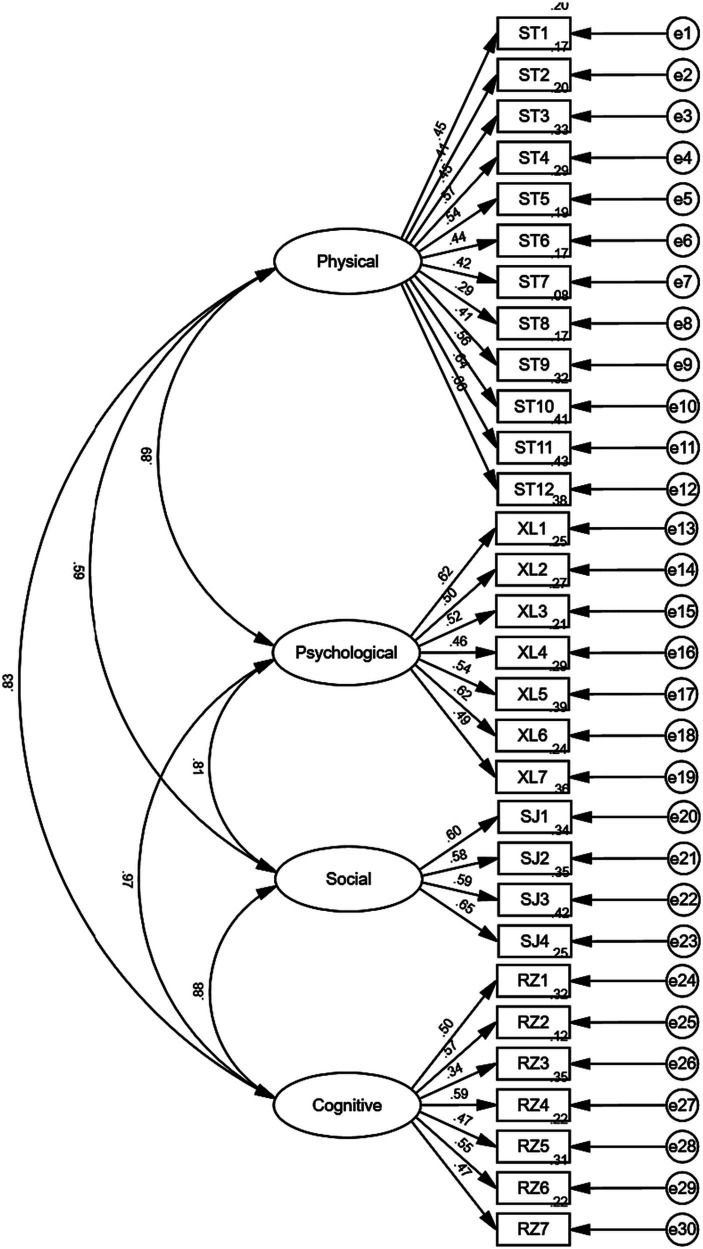
CFA model with item loadings.

The study conducted a validated factor analysis on 642 validated questionnaires. Inter factor path analysis and model fit tests were performed on the PL-C Quest (Chinese) using IBM SPSS Amos 26.0 software. The 30 items were categorized into four dimensions according to the theoretical framework of the scale, and the four-dimensional model of the scale was estimated. The model fit indices of the PL-C Quest (Chinese) were examined by using the chi-square degrees of freedom ratio χ^2^/df, RMSEA, CFI, IFI, and TLI, and the value of the chi-square degrees of freedom ratio χ^2^/df was 2.299, which was less than 3, indicating that the fit was good; The RMSEA was 0.043, less than 0.1, indicating a good fit. The coefficient values of CFI, IFI, and TLI were all above 0.9 and close to 0.9, resulting in a good fit, and the specific results of the fit are shown in Table 5. The path coefficients of the standardized output of the model are shown in Figure 3. Taken together, the dimensions of the model show strong correlations, and the factor loadings of the individual question items range from 0.29 to 0.66. Flexibility (0.29) and rule (0.34) have low factor loadings and factor loadings >0.4 for the remaining items are barely acceptable (Shearer et al., 2021).

**Table 5:** Model fit for PL-C Quest (Chinese) (*N* = 642).

CMIN/DF	RMSEA	CFI	TLI	IFI
<3	<0.08	>0.85	>0.85	>0.85
2.299	0.05	0.862	0.85	0.863

Root mean squared error of approximation (RMSEA); Comparative fit index (CFI); Tucker Lewis index (TLI); Incremental fit index.

## 4 Discussion

In this study, the PL-C Quest was revised for the first time to meet the cultural and psychometric standards of mainland China, and the generalizability of the questionnaire was tested. The overall results show that the PL-C Quest (Chinese) has good reliability. Comparison of reliability with the Australian original scale (n = 669, mean age of 10.1 years SD = 1.7) found that the reliability of the social domains appeared to be relatively low results <0.7 (AUS: retest reliability ICC = 0.66; CHN: Cronbach’s *α* = 0.65), and the other dimensions all showed results >0.7 in the excellent range (Barnett et al., 2022). However, the reliability results for all other dimensions were > 0.7 in the excellent range (Landis and Koch, 1977). It cannot be excluded that the social domain is a relatively small percentage of the overall scale (Li et al., 2020). PL-C Quest constructs measurement situations such as group activities and losing games to determine the development of children’s perceptions of winning and losing and inclusion behaviors. Such an assessment scenario would lead to different options so that consistency would be lower. Additionally, verified through split-half reliability (0.84) and McDonald’s omega (0.903) confirmed the overall reliability of the scale.

In the PL-C Quest (Chinese) validity test, item 8 in the physical domain (Flexibility 0.29) and item 3 in the cognitive domain (Rule 0.34) had poor factor loadings. We also checked the overall model coefficients by removing the two items with factor loadings <0.4 (Lloret et al., 2017), but the results were unchanged. Comparing the factor loading coefficients in the original scale, “Rules” was also a poor item (0.40), but Flexibility (0.45) met acceptable criteria (Barnett et al., 2022). In this regard, it was due to the interconnection between the domains and elements (Keegan et al., 2019). Since 2019, ASC has released APLF which describes the developmental model of physical literacy in 30 elements from 4 aspects, including physical, psychological, social, and cognitive (Australia Sport 2019). However, how to develop assessment tools compatible with APLF has been an essential issue that ASC is looking forward to solving. In the end, the PL-C Quest was derived from the APLF, and the 30 questions of the PL-C Quest corresponded to each of the 30 elements of physical literacy in the APLF (Australia Sport 2021), which scientifically enhances the overall level of children’s physical literacy by optimizing the correlation between the elements and thereby enhancing the different elements of children’s physical literacy (Barnett et al., 2023).

The results of the study show that four dimensions in the PL-C Quest (Chinese) CFA model have strong covariance (0.59 to 0.97), and the covariance value of the psychological and cognitive domains is close to 1, which is close to the results of the original scale (Barnett et al., 2022). This reflects the theoretical model based on physical literacy (Keegan et al., 2019), “Physical literacy is about helping people lead healthy and fulfilling lives through sport and physical activity through sustained changes in physical, psychological, cognitive, and social competencies.” Individuals with physical literacy can integrate their abilities to promote health and accomplish movement and physical activity throughout their lives” (Filho et al., 2021).

Since the announcement of the PL-C Quest in 2021 (Australia Sport 2021), only measures of Australian children (original scale) have been retrieved internationally. Compared with Australian children, Chinese children (Mean 96.76, SD 14.692) had overall higher levels of self-rated physical literacy than Australian children (Mean 93.5, SD 16.6) (Barnett et al., 2022). In the physical domain, Reaction time (56.6) was optimal for AUS children, and Agility (63.4) was optimal for CHN children. Interestingly, the poorer item in the physical domain for both AUS (23.3) and CHN (37.9) was Moving using equipment. Moreover, comparing the validity results, the factor loading for the item is relatively low (AUS 0.4 vs. CHN 0.408). The scenario for this item is “Child skateboarding well vs. child skateboarding poorly” (Australia Sport 2021). According to the definition of this question, “Movement skills used to move on, in or with equipment from one place to another” (Barnett et al., 2023), using single skateboarding/snowboarding as a substitute for moving equipment may overlook the importance of children’s skateboarding skills. Moving equipment may ignore children’s mastery of other moving equipment.

Turning back to the psychological and cognitive domains, the lower self-assessment items demonstrated strong cultural consistency. The AUS children all chose Confidence as the weaker item in the psychological domain (total 42.2, boys 17.7 and girls 16.7). The CHN children all chose Self-perception as the weaker item (a total of 20.4 of 8.4 boys, 16.4 girls). Furthermore, the weakest item in the cognitive domain for both AUS (11.8) and CHN (12.5) was Reasoning, which was defined as “Child looking outside at backyard in the rain and decides to play actively” Indoors vs. a child who is unsure what to do next” (Australia Sport 2021). According to the APLF’s definition of the item and analysis of the results (Barnett et al., 2023), the majority of children may lack motivation to play sports (the ability to change or justify beliefs about actions).

Strong cultural differences were seen in the social domain comparisons, with the most highly scored item in CHN (71), Ethics, being the weaker item for AUS children (9.6), and the most highly scored item in AUS children’s choice, Relationships (62.4), being the weaker item for CHN children (10.1). The scenario for Relationships was “Child offering for child to join ball game vs. child not welcoming child to join game.” The scenario for Ethics was “Child shakes hand to children at the end of the game vs. a child who walks away without shaking hands.” Surprisingly, when the assessor withdrew the PL-C Quest (Chinese), the children wrote on the scale that “when choosing relationships, it is not that I do not welcome other children to join in the game, but I am shy inviting them.” This result is highly relevant to the cultural context of China, where some studies have shown that Chinese parenting styles are more likely to lead to shyness in children (Liu et al., 2020). The result is also enough to illustrate the importance of scales that reflect children’s genuine internal emotions for children’s physical literacy or other health measures in various cultural contexts (Hulteen et al., 2020), providing ideas for scale improvement.

Previously, China’s use of the CAPL-2 to measure physical literacy in 8–12 children found that more than 80% of Chinese children were in the beginning and progressing stages (Li et al., 2020). That has led to the development of the CAEPL in China as part of the country’s policy on improving children’s physical literacy (Chen et al., 2020). However, it is necessary to optimize the assessment method and the specific implementation process in the future. First, it is necessary to get rid of the shackles of pure physical fitness testing, pay attention to children’s emotional and behavioral elements, and lead to the formation of an active physical literacy assessment system. Children are at the beginning of the journey of life, and their level of physical development only shows the period’s results; the movement’s emotion and cognition are the key to influencing the children’s lifelong active degree (Edwards et al., 2018). Therefore, to improve children’s physical literacy, it is necessary to start from the position of “body and mind” (Goss, 2021).

Overall, using the PL-C Quest to understand the physical literacy levels of children in different cultures can help educational policymakers around the world clarify their direction. The PL-C Quest (Chinese) survey was conducted with real feedback from many children. Moreover, a meta-analysis study comparing multiple physical literacy assessment tools showed that people with intellectual disability can also use the illustrated PL-C Quest (Barnett et al., 2023). In the future, it can test people with intellectual disability in China and other countries to cultivate their PL levels and provide valid evidence. Although this study provides essential validation evidence in cultural adaptation and reliability and validity tests, some aspects could be improved. For example, additional data representative of Chinese demographic variables (e.g., BMI, family background) need to be analyzed, as well as a survey of 4- to 5-year-old children—further psychometric evidence, such as evidence of measurement invariance and criterion validity.

## 5 Conclusion

This study introduced the PL-C Quest through translation, back-translation, presurvey, etc. The PL-C Quest (Chinese) aligns with mainland China’s linguistic expression, comprehension habits, and sociocultural background. The PL-C Quest (Chinese) stability was verified by analyzing the valid data of 642 children and comparing the results of the original scale. It was found that there was consistency in the weaker items of children’s perceived physical literacy across cultures, with more significant differences in the social domain. In the future, the test data of PL-C Quest among children worldwide should be supplemented to explore children’s self-reported levels of physical literacy, to stimulate children’s self-development and management, and to develop or promote more assessment scales that focus on children’s inner real feelings.

## Data availability statement

The raw data supporting the conclusions of this article will be made available by the authors, without undue reservation.

## Ethics statement

The study involving human participants were reviewed and approved by the Ethics Committee at Shanghai University of Medicine and Health Science (protocol code: 2023-PEEC-10-150203198809134265). The studies were conducted in accordance with the local legislation and institutional requirements. Written informed consent for participation in this study was provided by the participants' legal guardians/next of kin.

## Author contributions

YW: Conceptualization, Data curation, Writing – original draft, Writing – review & editing, Formal analysis, Software. XW: Writing – review & editing, Data curation, Formal analysis, Visualization. HW: Funding acquisition, Methodology, Writing – review & editing, Conceptualization, Project administration, Resources. LijW: Funding acquisition, Supervision, Writing – review & editing, Methodology. YT: Writing – review & editing, Formal analysis, Funding acquisition, Supervision. ZJ: Funding acquisition, Writing – review & editing, Validation. LiyW: Conceptualization, Funding acquisition, Project administration, Supervision, Validation, Writing – review & editing.
