# Supplementary material for: Validation of the PL-C Quest in China: understanding the pictorial physical literacy self-report scale
Source: Front Psychol. 2024 Mar 7;15:1328549. doi: 10.3389/fpsyg.2024.1328549 (PMC10956100; doi:10.3389/fpsyg.2024.1328549)
Supplement: Supplementary file 1 [file Data_Sheet_1.docx]

| Variable | Item | Item | CC value | T value | P value |
| --- | --- | --- | --- | --- | --- |
| Physical | Movement skills | 1 | .464** | 11.013 | <.001 |
|  | Moving using equipment | 2 | .445** | 12.975 |  |
|  | Object manipulation | 3 | .542** | 10.878 |  |
|  | Coordination | 4 | .537** | 15.615 |  |
|  | Stability/balance | 5 | .469** | 17.47 |  |
|  | Flexibility | 6 | .441** | 11.773 |  |
|  | Agility | 7 | .449** | 9.563 |  |
|  | Strength | 8 | .441** | 8.758 |  |
|  | Muscular endurance | 9 | .530** | 12.154 |  |
|  | Cardiovascular endurance | 10 | .587** | 14.776 |  |
|  | Reaction time | 11 | .594** | 16.219 |  |
|  | Speed | 12 | .622** | 17.315 |  |
| Psychological | Engagement enjoyment | 1 | .524** | 18.544 | <.001 |
|  | Confidence | 2 | .535** | 12.11 |  |
|  | Motivation | 3 | .492** | 12.499 |  |
|  | Connection to place | 4 | .564** | 13.506 |  |
|  | Self perception | 5 | .613** | 17.312 |  |
|  | Self regulation (emotions) | 6 | .489** | 15.293 |  |
|  | Self regulation (physical) | 7 | .518** | 9.289 |  |
| Social | Relationships | 1 | .483** | 12.126 | <.001 |
|  | Collaboration | 2 | .487** | 10.622 |  |
|  | Ethics | 3 | .543** | 10.423 |  |
|  | Society & culture | 4 | .518** | 12.152 |  |
| Cognitive | Content knowledge | 1 | .565** | 10.445 | <.001 |
|  | Safety & risk | 2 | .444** | 15.176 |  |
|  | Rules | 3 | .589** | 7.36 |  |
|  | Reasoning | 4 | .504** | 16.649 |  |
|  | Strategy & planning | 5 | .556** | 13.316 |  |
|  | Tactics | 6 | .480** | 14.894 |  |
|  | Perceptual awareness | 7 | .464** | 10.998 |  |

**Table 1 Total Item Analysis for PL-C Quest (Chinese) (N=642)**

CC value (correlation coefficient value) CR values（T value）
